# Antigen-specific immunotherapy combined with a regenerative drug in the treatment of experimental type 1 diabetes

**DOI:** 10.1038/s41598-020-76041-1

**Published:** 2020-11-03

**Authors:** Adrian Villalba, Silvia Rodriguez-Fernandez, David Perna-Barrull, Rosa-Maria Ampudia, Laia Gomez-Muñoz, Irma Pujol-Autonell, Eva Aguilera, Ruth M. Risueño, Mary Cano-Sarabia, Daniel Maspoch, Federico Vázquez, Marta Vives-Pi

**Affiliations:** 1 Immunology Section, Germans Trias i Pujol Research Institute, Autonomous University of Barcelona, Carretera Canyet s/n, 08916 Badalona, Spain; 2 Endocrinology Section, Germans Trias i Pujol Research Institute, Autonomous University of Barcelona, Badalona, Spain; 3 Josep Carreras Leukaemia Research Institute, Campus GTP-ICO, Badalona, Spain; 4 Catalan Institute of Nanoscience and Nanotechnology, CSIC, The Barcelona Institute of Science and Technology, Bellaterra, Spain; 5 ICREA, Pg. Lluís Companys 23, 08010 Barcelona, Spain

**Keywords:** Immunology, Endocrinology

## Abstract

Type 1 diabetes is an autoimmune disease caused by the destruction of the insulin-producing β-cells. To revert type 1 diabetes, the suppression of the autoimmune attack should be combined with a β-cell replacement strategy. It has been previously demonstrated that liraglutide, a glucagon-like peptide-1 receptor agonist, restores β-cell mass in type 1 diabetes, via α-cell transdifferentiation and neogenesis. We report here that treatment with liraglutide does not prevent type 1 diabetes in the spontaneous non-obese diabetic (NOD) mouse model, but it tends to reduce leukocytic islet infiltration. However, in combination with an immunotherapy based on tolerogenic liposomes, it is effective in ameliorating hyperglycaemia in diabetic NOD mice. Importantly, liraglutide is not detrimental for the tolerogenic effect that liposomes exert on dendritic cells from patients with type 1 diabetes in terms of membrane expression of molecules involved in antigen presentation, immunoregulation and activation. Moreover, the in vivo effect of the combined therapy was tested in mice humanised with peripheral blood mononuclear cells from patients with type 1 diabetes, showing no adverse effects in leukocyte subsets. In conclusion, the combination therapy with liraglutide and a liposome-based immunotherapy is a promising candidate strategy for type 1 diabetes.

## Introduction

Type 1 diabetes (T1D) is a metabolic disease caused by the autoimmune destruction of the insulin-producing pancreatic β-cells. Currently, there is no cure nor prevention for the disease and subjects with T1D need exogenous insulin administration to survive. However, this treatment does not lead to continuous normoglycaemia and may often produce events of hyper- and hypoglycaemia. Patients with T1D display important associated complications—such as neuropathy, nephropathy and retinopathy—that, together with episodes of glycaemia dysregulation, worsen their quality of life and may shorten their lifespan^1^.

At present, single immunotherapies that have been successful in experimental models have failed in humans^2^. On the other hand, autologous regenerative strategies, by recovering β-cell mass, are not able to ameliorate the disease due to the chronic autoimmune process. Given that, there is an urgent need for novel therapies combining the regeneration of endogenous β-cells and the arrest of the autoimmune reaction against β-cells. Combined therapies using different strategies must be designed for human use. There is a clinical trial already completed in humans^3^, which consists of the administration of GAD-aluminium conjugate as immunotherapy^4^ and GABA^5^ as a regenerative agent (NCT02464033). This resulted in a decrease in daily insulin requirements from baseline to 12 months and the improvement of residual β-cell function by means of C-peptide secretion (a product of proinsulin processing). Additionally, a more recent combined strategy—consisting of anti-IL21 and liraglutide—resulted in the restoration of normoglycaemia in diabetic mice^6^. On the one hand, the administration of anti-IL21 achieved normoglycaemia in some diabetic mice. On the other hand, liraglutide, a commercially available drug for type 2 diabetes, contributed to the recovery of the β-cell mass. Because anti-IL21 has systemic effects in the immune system, antigen-specific immunotherapies could be safer and help to overcome the side-effect issues derived from total immunosuppression. All these data indicate that combined therapies both targeting the autoimmune attack and promoting β-cell regeneration could result in a greater beneficial outcome for the treatment of T1D.

Our previous work demonstrated the ability of apoptotic β-cells to induce self-tolerance through efferocytosis by dendritic cells (DCs), thus preventing T1D in the non-obese diabetic (NOD) mouse model^7^. For clinical application, β-cell apoptotic cells were mimicked using nanotherapeutic tools. Liposomes, lipidic vesicles with an aqueous core, were designed with phosphatidylserine (PS) in the membrane and encapsulating insulin A and B chains (PSAB-liposomes). PSAB-liposomes prevented T1D in the NOD mice^8,9^ by inducing tolerogenic DCs. Furthermore, this tolerogenic effect was also validated in human DCs from subjects with T1D^10,11^. With the aim to combine this immunotherapy with a regenerative strategy, we previously performed a Drug Repurposing analysis to search for already-existing compounds able to promote β-cell regeneration. Drug Repurposing has the advantage to propose novel uses for compounds existing in the market and overcomes the safety issues. The analysis identified liraglutide, an agonist of GLP1 (aGLP1), as a regenerative drug^12^. In fact, liraglutide administration ameliorated hyperglycaemia in immunodeficient NOD-Scid IL2rg^-/-^ (NSG) mice rendered diabetic by Streptozotocin. This improvement correlated with the appearance of bihormonal insulin^+^glucagon^+^ cells and insulin^+^ cells in the ductal areas^12^. Previous studies demonstrated the ability of aGLP1 to induce transdifferentiation of glucagon-producing α-cells into β-cells^13^. Furthermore, aGLP1 induce β-cell proliferation through the inhibition of *DYRK1A* gene expression^14^. Ongoing clinical trials aim to assess the effect of liraglutide in subjects with T1D (NCT02617654 and NCT02516657).

Taking all this information into account, an experimental combined therapy consisting of an immunotherapy based on PSAB-liposomes to arrest autoimmunity and liraglutide to promote β-cell regeneration was designed. Here we show that this strategy is able to ameliorate hyperglycaemia in the spontaneous model of autoimmune T1D, the NOD mice. Importantly, we also show for the first time that liraglutide does not alter the tolerogenic effect of the immunotherapy in DCs from human adult patients with T1D. Additionally, the combined therapy does not appear detrimental for T lymphocyte subsets in a humanised mouse model. Investigation of combined therapies to restore tolerance and to allow the reestablishment of β-cell mass should continue to completely reverse T1D in a clinical setting.

## Results

### Liraglutide accelerates the onset of T1D in NOD mice

**Figure 1:**
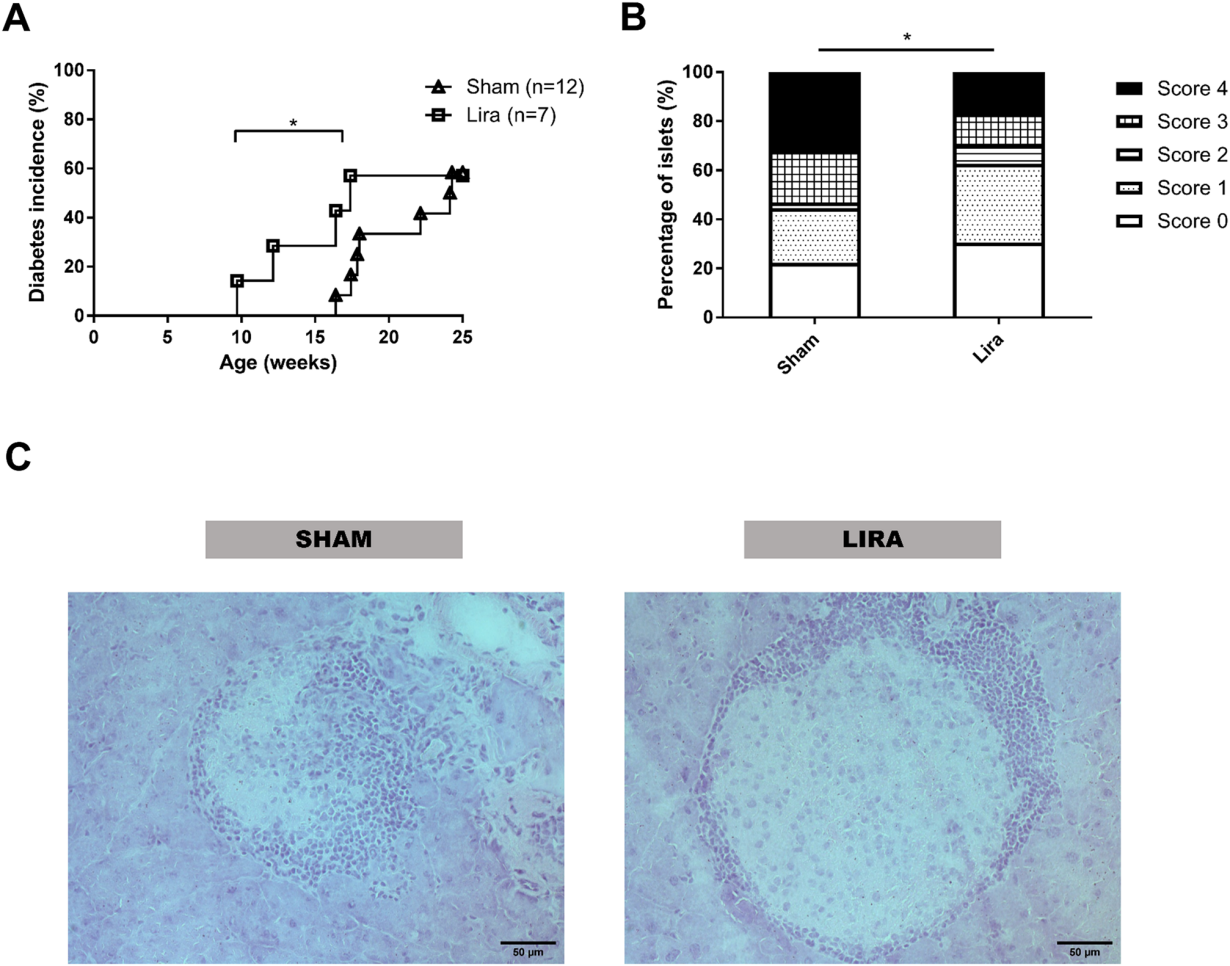
Effect of liraglutide in pre-diabetic NOD mice. **(A)** Incidence of diabetes (%) in NOD mice (female) treated with liraglutide (Lira, squares, n = 7) or PBS (Sham, triangles, n = 12). No significant differences in T1D incidence were found between groups (Mantel-Cox Log-Rank). Significant differences in the age of the onset were found between Lira and Sham groups (*p < 0.05, Mann–Whitney test). (**B)** Percentage of islets in each of the infiltration categories, in sham and liraglutide treated groups (n = 3/group): White = 0, no insulitis; Dotted = 1, peri-insular; Striped = 2, mild insulitis (< 25% of the infiltrated islet); Squared = 3, 25–75% of the islet infiltrated; Black = 4, > 75% islet infiltration. Significant differences were found between groups (*p < 0.05, Chi-square test). (**C)** Representative images of insulitis in islets from H/E-pancreatic cryostat sections (5 μm) from the two groups. Scale bars represent 100 μm.

Liraglutide has a regenerative effect on β-cells, which might alter the development of T1D. To assess their impact on the onset of the disease, NOD mice (n = 7) were treated with three doses of 1 mg/kg of liraglutide on alternate days during the prediabetic period (8 weeks old). Sham group (n = 12) was treated with PBS. liraglutide treatment significantly accelerated T1D onset when compared to sham group. Mice from the sham group developed the disease from the age of 16.4 weeks and with an incidence of 58.33% at 25 weeks of age. Treated mice developed T1D starting at 9.71 weeks of age and reaching an incidence of 57.14% at 25 weeks of age (Fig. 1A). No significant differences in terms of incidence were found between both groups at the end of the experiment.

Insulitis score was determined at the end of the follow-up period in non-diabetic mice. In mice treated with liraglutide, 62% of the islets remained non-destructed –insulitis free or with peri-insulitis—, whereas only 40% of the islets were non-destructed in the sham group (Fig. 1B,C). These differences are statistically significant, pointing to a strong difference in the proportion of each type of insulitis between treated and non-treated animals. Altogether, this data supports the hypothesis that a regenerative agent may accelerate T1D onset by exacerbating the autoimmune reaction.

Given that liraglutide can induce the formation of pseudoislets arising from the ducts ^12^, hereinafter called neoislets, insulitis score was determined for both neoislets and mature islets. Neoislets show an increased insulitis score in mice treated with liraglutide when compared to control mice, probably promoted by the β-cell regenerative effect of liraglutide in the ducts. On the opposite side, mature islets remain less affected by insulitis in liraglutide-treated mice than in controls (Supp. Figure 1).

### Liraglutide combined with liposome-based immunotherapy ameliorates hyperglycaemia in NOD mice

**Figure 2:**
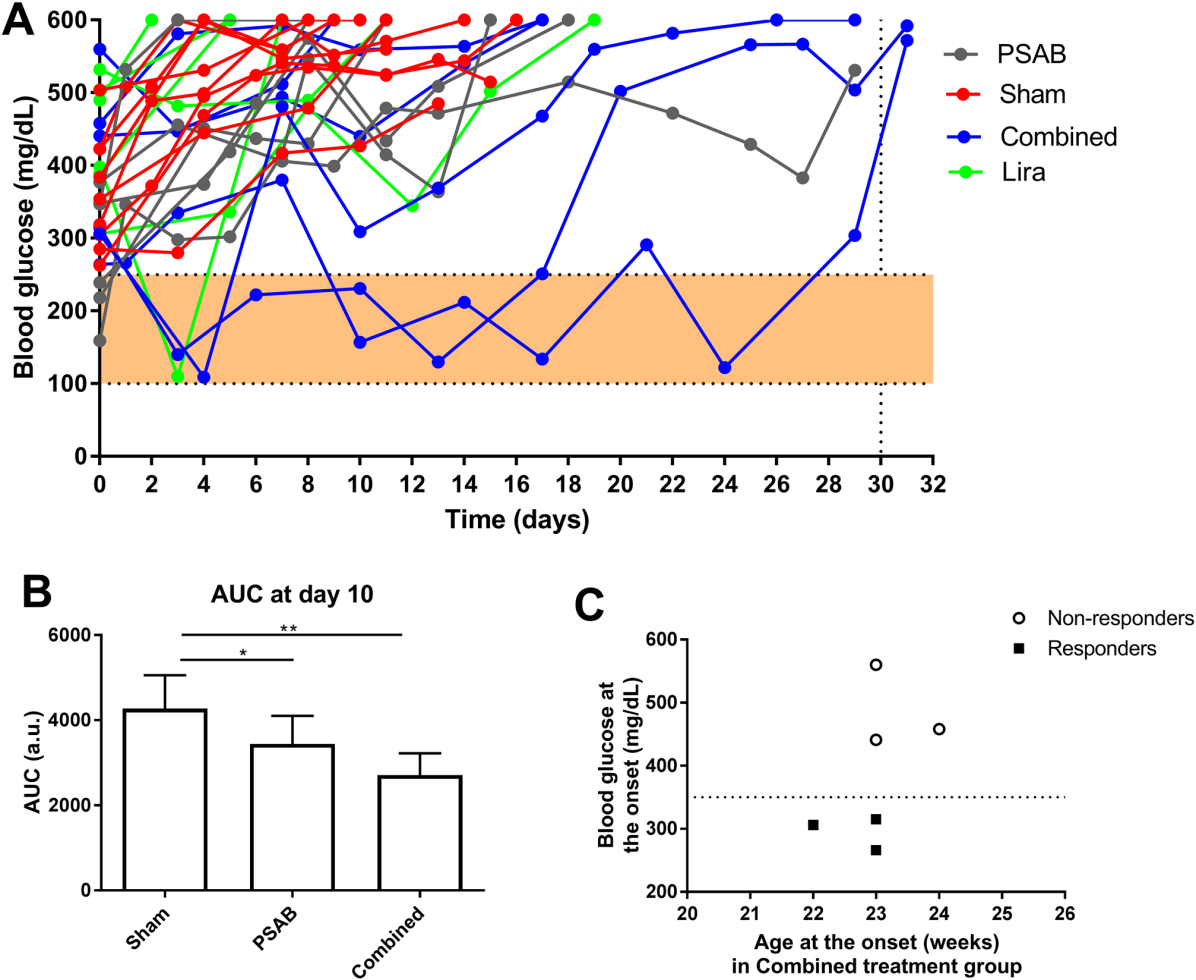
Effect of liraglutide in diabetic NOD mice. **(A)** 2 h fasting blood glucose levels (mg/dL) in diabetic NOD mice treated with either PBS (Sham, red, n = 9), PSAB-liposomes (PSAB, grey, n = 6), liraglutide (Lira, green, n = 6) or combined (PSAB + Lira, blue, n = 6). Filled area corresponds to normal blood glucose levels in NOD mice. **(B)** Area Under the Curve (AUC) of the graph in **(A)** at day 10, when all mice of Sham, PSAB, and combined groups remain alive. Only responder mice are represented in the combined group. Results are expressed as mean ± SD. Differences were found between Sham and PSAB (*p < 0.05, Mann–Whitney test) and between Sham and combined therapy (**p < 0.01, Mann–Whitney test). (**C)** Stratification of responders (black squares) and non-responders (white circles) in the group treated with combined therapy regarding the age (weeks) and blood glucose levels (mg/dL) at the onset.

Our previous results on the effect of liraglutide in regenerating β-cell mass^12^ led us to combine this drug with a PS-liposome based immunotherapy, which restores β-cell tolerance. NOD mice were assigned to treatment groups—sham, liraglutide, liposomes or combined—as soon as T1D was detected (Fig. 2A). Age and glycaemia at T1D onset were similar between groups and all mice included in the experiment developed T1D between 12 and 25 weeks of age. Liraglutide monotherapy did not ameliorate hyperglycaemia in mice with T1D, despite one mouse transiently achieved normoglycaemia at day 3 and immediately returned to hyperglycaemia. PSAB-liposome immunotherapy partially ameliorated blood glucose levels, but glycaemia was always maintained higher than 270 mg/dL. Importantly, the combined therapy resulted in a significant amelioration of glycaemia in 3 of 6 NOD mice (both the responders and non-responders are presented in the graph). Moreover, this group has a significant reduction on the glycaemia area under the curve when compared with sham and PSAB groups (Fig. 2B), correlating with an improvement in survival compared to the sham group and group treated only with PSAB-liposomes. These responder mice showed a tendency, although non-significant, to display lower blood glucose levels at T1D onset when compared to non-responders (Fig. 2C).

### Liraglutide does not interfere with the tolerogenic ability of liposomes in human DCs

**Figure 3:**
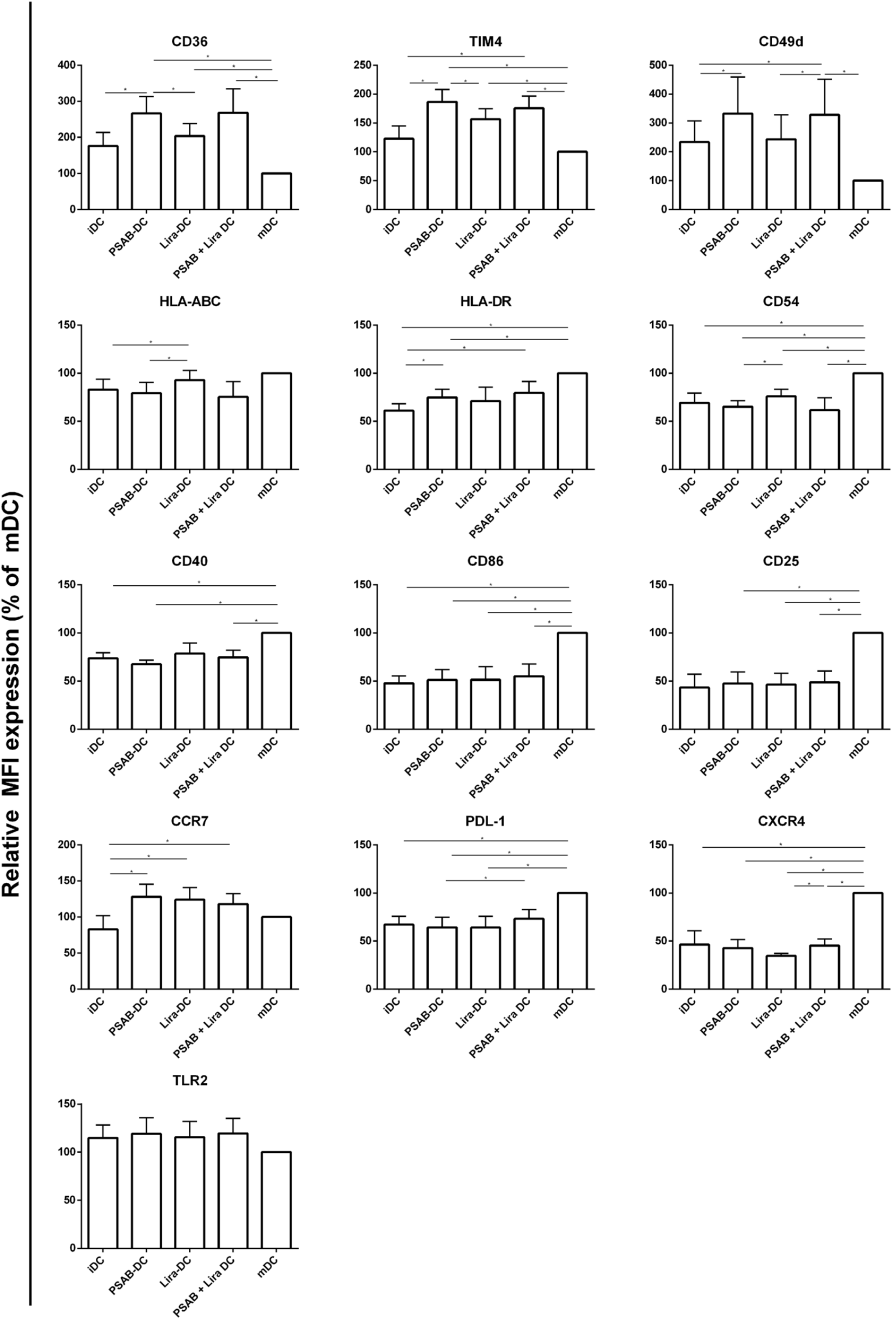
Combined therapy regulates dendritic cell (DCs) phenotype. Relative CD36, TIM4, CD49d, HLA-ABC, HLA-DR, CD54, CD40, CD86, CD25, CCR7, PDL-1, CXCR4 and TLR2 membrane expression in DCs (n = 7) obtained from adult subjects with T1D. Results are mean ± SD. Significant differences were found when comparing culture conditions (*p ≤ 0.05, **p < 0.01, ***p < 0.001, ****p < 0.0001, Mann–Whitney test).

In order to identify possible interactions between the two parts of the combined therapy, DCs from patients with T1D were exposed to liraglutide and to PS-liposomes and analysed by flow cytometry (Supp. Figure 2). As expected, iDCs acquired a tolerogenic phenotype when treated with PSAB-liposomes (Fig. 3) in terms of CD36, TIM4, CD49d, HLA Class I, HLA Class II, CD54, CD40, CD86, CD25, CCR7, PD-L1, CXCR4, and TLR2, in comparison to mDCs. Liraglutide alone was not able to modify iDC membrane expression of these molecules except for an increase in HLA Class I and CCR7. Importantly, the treatment of DCs with the combination of liraglutide and PSAB-liposomes resulted in a phenotype identical to tolerogenic treatment with PSAB-liposomes in terms of CD36, TIM4, CD49d, HLA Class I, HLA Class II, CD54, CD40, CD86, CD25, CCR7, CXCR4, and TLR2. Interestingly, liraglutide in combination with PSAB-liposomes increased the expression of PD-L1 in DCs when compared to PSAB-liposomes monotherapy, reinforcing the tolerogenic potential of DCs. These changes were predominantly driven by PSAB-liposomes due to their tolerogenic potential as described^10^. Of note, PSAB-liposomes alone induce changes in the membrane expression of these molecules, even in absence of liraglutide, that support a tolerogenic role when compared to mDCs and iDCs.

### The transcriptional changes induced in DCs from patients with T1D by the combined therapy point to a tolerogenic effect

**Figure 4:**
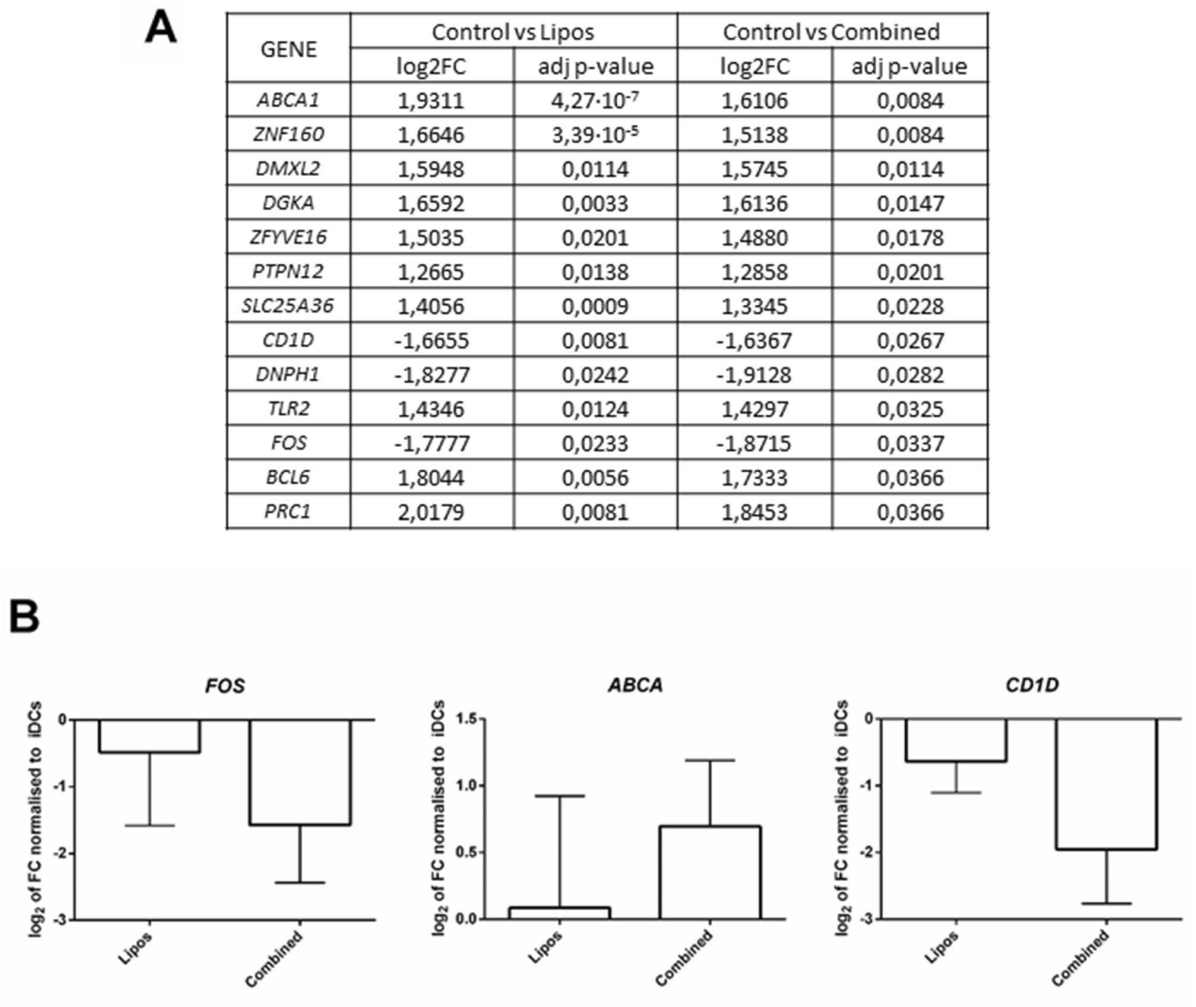
Gene expression analysis of human DCs co-cultured with PSAB-liposomes and combined therapy. **(A)** List of differentially expressed genes in both PSAB and combined groups in comparison to control conditions, showing log_2_ of Fold Change and adjusted p-value. (**B)** Relative gene expression of 3 selected genes from A) (FOS, ABCA, CD1D) analysed by qRT-PCR. Gene expression was normalized to *GAPDH*. Bars show the mean ± S.D. of the Log2 of FC using basal transcription as standard value (Wilcoxon test). Validation of the differential expression of three selected genes from A) (*FOS*, *ABCA*, *CD1D*) by quantitative RT-PCR (n = 3). Results are mean ± SD.

In order to determine if liraglutide altered the previously described tolerogenic effect of PSAB-liposomes in human DCs^10^, RNA-seq analysis was performed in DCs from four patients with T1D. Their phagocytosis capability was confirmed by flow cytometry using fluorescent liposomes. After 4 h of co-culture, 72.83 ± 9.27% (mean ± SD) of DCs were positive for fluorescent signal. RNA integrity was determined for each sample, RIN 9.38 ± 0.23 (mean ± SD) being optimal for RNA-seq experiment. Bioinformatics analysis of the RNA-seq revealed 179 differentially expressed genes when comparing iDCs and PSAB-DCs (adjusted *p* value < 0.05 and Log2 of fold change > or < 1.2). Of these 179 genes, 142 (79.33%) were downregulated and the remaining 37 (20.67%) were upregulated, and 152 corresponded to protein-coding genes. The comparison between iDCs and combined therapy (liraglutide + PSAB-liposomes) displayed 24 differentially expressed genes (adjusted *p* value < 0.5). Of these 24 genes, 7 (29.16%) were downregulated and the remaining 17 (70.84%) were upregulated, and finally 21 corresponded to protein-coding genes. In both cases, the gene expression was modulated toward a similar profile in DCs exposed to both conditions. Taking into account the genes with identical altered expression were found in both analysis (Fig. 4A), three of them were validated by qRT-PCR (Fig. 4B).

### The combined therapy does not alter human leukocyte subsets in humanised mice

**Figure 5:**
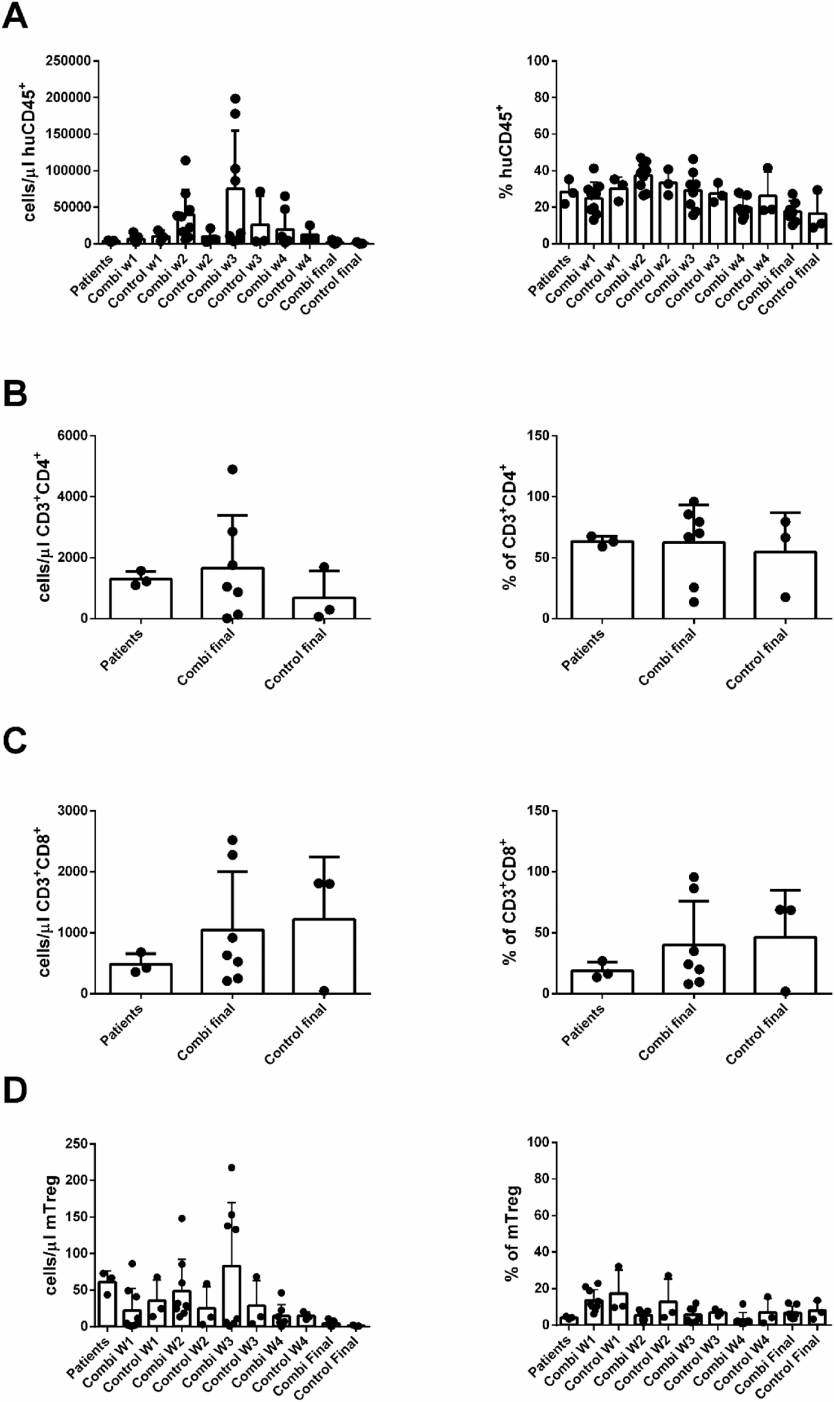
In vivo effect of the combined therapy in PBMC-humanised NSG mice. Total counts (left) and percentage (right) of human cells at the starting point (in patients), in mice from week 1 (w1) to week 4 (w4) post-transplantation and at the endpoint (day 30 of treatment, final). (**A)** Data of human CD45^+^ cells. (**B)** Data of human CD3^+^CD4^+^ cell subset. (**C)** Data of human CD3^+^CD8^+^ cell subset. (**D)** Data of human memory T regulatory cells (mTreg) subset, identified as CD3^+^CD4^+^CD127^low^CD25^+^CCR4^+^CD45RO^+^. Data refers either to patients (n = 3), combined (n = 7) or control (n = 3) group.

To further investigate the short-term effects of the combined therapy in human leukocyte subsets, NSG mice humanised with PBMCs from patients with T1D were exposed to the combined therapy and monitored for 4 weeks. No alterations in leukocyte counts or percentage were found when comparing treated mice to control group (Fig. 5A). The number and percentages of T lymphocytes (both CD4^+^ and CD8^+^) were not altered by the combined therapy (Fig. 5B,C). Similarly, the number and percentages of memory regulatory T cells (mTreg) cells were similar to the control group during the follow-up (Fig. 5D).

## Discussion

The present study shows that a combined therapy consisting of a regenerative drug and a liposome-based immunotherapy—PSAB-liposomes and liraglutide—can ameliorate hyperglycaemia in the spontaneous model of autoimmune diabetes, the NOD mouse. In fact, 50% of the treated animals respond to the treatment. First, the effects of liraglutide as a regenerative agent in autoimmune diabetes were assessed by its administration in prediabetic NOD mice. Liraglutide administration resulted in an acceleration of the onset but without altering the incidence of diabetes. These results fit well with a previous study showing that liraglutide did not prevent or delay the onset of the disease in NOD mice^6^. It is reasonable to speculate that the here reported acceleration of T1D onset can be due to the increase in β-cell autoantigens exposure/release induced by liraglutide in a model with active autoimmunity. This could be due to the fact that neoislets increase the availability of autoantigens—at least insulin^12^—that accelerate the autoimmune attack and actively recruit lymphocytes. Overall, these data would not only explain the acceleration in the onset of diabetes but also suggest that regenerative agents like liraglutide are not efficient in blocking an autoimmune attack and thus would need to be combined with an immunotherapy to restore β-cell tolerance in T1D. An interesting point is that previous studies with combined therapies including liraglutide did not observe β-cell proliferation^6^, but our previous results demonstrated that liraglutide is involved in β-cell replacement, mainly by induction of bihormonal (glucagon^+^insulin^+^) cells and from insulin-producing cells in the ducts^12^.

In view of this, a therapy was designed by combining the administration of PSAB-liposomes to arrest autoimmunity^8,9^ prior to liraglutide treatment to promote β-cell regeneration^12^ in diabetic NOD mice. This strategy resulted in the amelioration of hyperglycaemia in 50% of the treated mice, with some even reaching normoglycaemia. The improvement in the blood glucose levels was transient and mice became hyperglycaemic again after the withdrawal of liraglutide. This phenomenon was previously observed upon liraglutide administration in diabetic immunodeficient mice^12^, suggesting that this drug may be necessary for maintaining physiological β-cell function. However, it would be of interest to explore further modifications of the current approach such as dosages and administration time-points. Intriguingly, the stratification between responders and non-responders showed that those developing T1D with lower glycaemia at the onset were prone to be responders independently of the age. This may indicate that, as proposed^15^, residual β-cell function is a key feature to be considered in the design of combined therapies. It would be of relevance to further expand these observations to other models of autoimmune diabetes, like the inducible RIP-B7.1 mouse model^16^.

Hence, to determine if liraglutide affected the immunoregulatory effects of PSAB-liposomes, DCs from human subjects with T1D were exposed in vitro to both products. In summary, PSAB-liposomes can induce a tolerogenic phenotype in DCs on their own and even combined with liraglutide. This is relevant data because it demonstrates that liraglutide does not alter the tolerance achieved by PSAB-liposomes phagocytosis in DCs. As expected, liraglutide per se was not able to induce phenotypic alterations in iDCs. This is a particular feature of the combined therapy since the regenerative agent does not interfere with the immunotherapy in terms of the arrest of autoimmunity. The question about liraglutide affecting the tolerogenic properties of DCs treated with PSAB-liposomes was also addressed at the gene expression level. RNA-seq experiments revealed that the main gene alterations related to tolerance were found in DCs both with and without liraglutide treatment. Further research is still needed at the transcriptomics level to find a robust and strong genetic signature by both increasing the sample size and by exploring different time-points of co-culture.

Finally, the establishment of a humanised mouse model by injecting PBMCs from adult subjects with T1D in immunocompromised NSG mice enabled us to study the effect of the combined therapy in a broader range of human immune cells. No detrimental effect of the combined therapy in the different subsets of human T lymphocytes was detected. The poor, almost null, engraftment of B lymphocytes and NK cells limited the outcome of this experiment. It would be interesting to evaluate the combined therapy in other humanisation models, especially if they can develop diabetes, to achieve a clearer picture of the effects of the combined therapy and smooth its way for a clinical translation.

Currently, there are other combined therapies in the experimental field comprising liraglutide as a regenerative drug^6^. The main disadvantage in this approach is the use of anti-IL21 as immunotherapy, given that it is a systemic immunosuppressive agent. Other immunosuppressive agents totally ablating the immune system have proven useful in the arrest of autoimmunity in T1D and even in the reversal of the disease^17,18^, but one would have to assume the side effects related to immunosuppression as well^19^. In that case, it would be much more advantageous to focus on antigen-specific therapies targeting specifically the autoimmune reaction against the insulin-producing β-cells.

Further studies are required to ameliorate and fully characterise the effect of the here presented combined therapy. New schedules of administration should be explored in order to gain insights into the amelioration of hyperglycaemia upon different dosages and time-points of treatment. Additionally, it would be of interest to evaluate the effect of the combined therapy in other murine models of the disease, such as those induced by diabetogenic T cell transfer^20^.

In conclusion, the combination therapy consisting of PSAB-liposomes and liraglutide is a strategy that can ameliorate hyperglycaemia in half of the mice with overt T1D. Moreover, the regenerative effect of liraglutide does not interfere with the tolerogenic consequences of PSAB-liposomes in the human immune system. Despite additional research is still needed to explore the regenerative potential of liraglutide, the combined therapy could be useful for the treatment of T1D.

## Methods

### Liposome manufacturing

Liposomes consisted of 1,2-dioleoyl-sn-glycero3-phospho-L-serine (sodium salt) (DOPS, Lipoid, Steinhausen, Switzerland) and 1,2-didodecanoyl-sn-glycero-3-phosphocholine (DLPC, Lipoid), and cholesterol (CH, Sigma Aldrich, Saint Louis, MO, USA). Liposomes were generated encapsulating the murine or human insulin A and B chains (PSAB-liposomes) (Table 1) using the thin film hydration method from the lipid mixture, at a final lipid concentration of 30 mM, as described^8–10^.

**Table 1:** Features of the liposomes used in the study.

Liposome	Peptide	Specie	Diameter (nm)	Polydispersity index (PdI)	ζ-potential (mV)	Encapsulation (%)
mPSA-lipos	Insulin_90–110_ (A chain)	*Mus musculus*	712	0.364	− 46.6	32.29
mPSB-lipos	Insulin_25–54_ (B chain)	*Mus musculus*	628	0.325	− 44.9	89.63
hPSA-lipos	Insulin^a^ (A chain)	*Homo sapiens*	690 ± 29	0.40 ± 0.28	− 38.57 ± 6.76	39.74 ± 22.10
hPSB-lipos	Insulin^a^ (B chain)	*Homo sapiens*	788 ± 264	0.52 ± 0.42	− 37.50 ± 7.16	93.19 ± 0.92

The subindex refers to the position of the query sequence to the protein. Data are expressed as mean ± SD.

*PS* phosphatidylserine, *A* insulin A chain, *B* insulin B chain.

^a^These peptides correspond to the whole A or B chains of the insulin peptides.

### Mice and treatment

NOD mice were bred in our own facility and kept under specific pathogen-free conditions in a 12 h dark/12 h light cycle provided with food and water ad libitum. Mice with either successive 2 h fasting blood glucose levels higher than 250 mg/dl or with a measure higher than 300 mg/dl were considered diabetic. For prevention experiments, prediabetic NOD females—8 weeks-old—were injected with 3 doses of either PBS (Sham) or liraglutide (Lira, 1 mg/kg of body weight) biweekly. For treatment experiments, NOD mice with overt diabetes—12 to 25 weeks-old—were treated daily s.c. only with liraglutide throughout the experiment (Lira, n = 6), only with i.p. PSAB-liposomes at days 1, 3 and 7 (PSAB, n = 6), combining i.p. PSAB-liposomes at days 1, 3 and 7 with daily s.c. liraglutide from day 4 to 30 (combined group) (PSAB + Lira, n = 6). A sham group was included by treating mice daily s.c. with PBS (Sham, n = 6). Mice were monitored biweekly for fasting glucose levels. Mice were euthanised when blood glucose was higher than 600 mg/dL for ethical reasons. At the end of the follow-up, mice were euthanised by cervical dislocation and pancreases were harvested and snap-frozen in an isopentane/cold acetone bath.

### Insulitis score

Pancreases from non-diabetic animals (n = 3) of the incidence study at 25 weeks of age were used to determine the insulitis score. Non-overlapping cryosections of 5 μm were obtained and stained with haematoxylin and eosin. A double-blind analysis was performed by independent observers. A minimum of 40 islets per animal was scored to determine the degree of leukocyte infiltration (insulitis), as previously described ^21^: 0, no insulitis; 1, peri-insular; 2, mild insulitis (< 25% of the islet infiltrated); 3, 25–75% of the islet infiltrated; 4, > 75% islet infiltration.

### Patients

Adult patients with T1D (n = 13, Table 2) were included in the study. All patients fulfilled the diagnosis criteria for T1D. The inclusion criteria were 18–45 years of age and a normal body mass index (BMI, 18.5–30 kg/m^2^). Exclusion criteria were being under immunosuppressive or anti-inflammatory treatment, or undergoing pregnancy. All study participants gave informed consent, and the study was approved by the Committee on the Ethics of Research of the Germans Trias i Pujol Hospital.

**Table 2:** Data from the patients included in the peripheral blood collection.

N	Gender (M/F)	Age (years)	BMI	Age at onset (years)	HbA1c (%)
13	3/13	26.7 ± 4.5	22.2 ± 2.4	13.9 ± 9.6	7.3 ± 0.9

Data presented as mean ± SD.

*BMI* body mass index.

### Generation of human DCs

Peripheral blood mononuclear cells (PBMCs) were obtained from 50 ml blood samples of adult patients with T1D (n = 7) after Ficoll Paque density gradient centrifugation (GE Healthcare, Marlborough, USA). Monocytes were isolated using the EasySep Human CD14 Positive Selection Kit (STEMCELL Technologies, Vancouver, Canada) and cultured with IL-4 and GM-CSF as described ^10^ to derive DCs. After 6 days of culture, DC differentiation yield was assessed by CD11c-APC staining (Immunotools, Friesoythe, Germany) using flow cytometry (FACS Canto II, BD Biosciences, San Jose, USA). The negatively selected fraction of PBMCs was cryopreserved in Foetal Bovine Serum (ThermoFisher Scientific, Waltham, MA, USA) with 10% dimethylsulfoxide (Sigma-Aldrich).

### Effects of the combined therapy in human DCs

DCs from patients with T1D (n = 7) were co-cultured with 1 mM PSAB-liposomes (PSAB-DC), 1000 nM Lira (Lira-DC) or combined (PSAB + Lira DC) for 24 h in the presence of 20 μg/ml human insulin (Sigma-Aldrich). DCs were cultured with 20 μg/ml human insulin (Sigma-Aldrich) to obtain immature DCs (iDCs) and adding a cytokine cocktail [1000 IU/ml TNFα and 2000 IU/ml IL-1β (Immunotools) and 1 μM Prostaglandin E_2_ (Cayman Chemical, Ann Arbor, USA)] to obtain mature DCs (mDC). To assess DCs phenotype, CD25-PE, CD86-FITC, HLA ABC-FITC, HLA DR-FITC, CD14-PE and CD40-APC (Immunotools), CD36-APCCy7, TIM4-APC, CD54-PECy7, TLR2-FITC, CXCR4-APCCy7, CCR2-APC, PD-L1-PECy7, ILT3-PECy7 (Biolegend, San Diego, USA) and CCR7-PECy7 (BD Biosciences) monoclonal antibodies were used to determine their membrane expression. All DCs conditions from the same patient were analysed at the time. All MFI groups were normalized to the MFI of mDCs.

### RNA-seq

DCs from four patients with T1D were cultured in basal conditions (iDCs), with 1 mM PSAB-liposomes (PSAB-DC), 1000 nM Lira (Lira-DC) or combined (PSAB + Lira DC) for 4 h. Cells were then harvested from culture wells with Accutase (eBioscience, San Diego, CA, USA). Viability and DC percentage were assessed by flow cytometry (FACS Canto II, BD Biosciences) after staining with 7aad (BD Biosciences), annexin V-PE and CD11c-APC (Immunotools). RNA was obtained using the RNeasy Micro Kit (QIAGEN, Hilden, Germany) following the manufacturer’s instructions, and RNA integrity was assessed by capillary electrophoresis in the TapeStation 2200 instrument (Agilent Technologies Inc., Santa Clara, CA, USA). Thus, 500 ng of total RNA was used to prepare the library. Ribosomal RNA (rRNA) was depleted through oligo-dT binding (mRNA Magnetic Isolation, New England Biolabs). Strand-specific libraries were made using the NEBNext Ultra II Directional RNA LibPrep kit (New England Biolabs), and the quality was evaluated again with TapeStation 2200 (Agilent Technologies Inc), and quantity, with KAPA Library quantification kit (Roche, Mannheim, Germany). RNA sequencing was performed using an Illumina sequencer (Illumina, San Diego, CA, USA) in a sequencing-by-synthesis protocol consisting of 2 × 75 cycles with 20 million reads. The differential gene expression analysis was performed using the DESeq2 algorithm^22^. Genes with an adjusted p-value < 0.5 and Log2 of fold change > 1.2 were considered upregulated, whereas those with Log2 of fold change < 1.2 were considered downregulated. Experimental data have been uploaded into the European Nucleotide Archive (EBI, https://www.ebi.ac.uk/ena; accession number: GSE144348). Confirmation through qPCR was performed in an independent cohort of 3 patients as previously described ^10,11^.

### Humanised mice

Normoglycaemic immunodeficient NSG mice at 8 weeks of age were humanised i.p. with 10^7^ PBMCs from three different adult subjects with T1D. PBMCs were obtained Ficoll Paque density gradient centrifugation (GE Healthcare) as aforementioned. At day 13 after injection, a blood sample (50 µl) was obtained from the tail of each mouse and stained for both human and murine CD45 (BD Biosciences). All mice with more than 10% of human/murine CD45 chimerism at day 13 were injected either with PBS (n = 3) or the combined therapy (n = 7). Then, a blood sample (50 µl) was obtained at each checkpoint and stained as previously described^23^. The percentage and count of memory regulatory T cells (mTregs, CD3^+^CD4^+^CD127^low^CD25^+^CCR4^+^CD45RO^+^) were analysed weekly throughout the treatment, and total T lymphocyte subsets were analysed at endpoint by flow cytometry from peripheral blood samples.

### Statistical analysis

Prism 7.0 software (GraphPad software Inc., San Diego, USA) was used to perform the statistical analysis. For comparisons of unpaired data, a non-parametric Mann–Whitney test was used and for paired comparisons, a non-parametric Wilcoxon test was used.

### Ethics statement

This study was carried out in strict accordance with the recommendations in the Guide for the Care and Use of Laboratory Animals of the Generalitat de Catalunya, Catalan Government. The protocol was approved by the Committee on the Ethics of Animal Experiments of the Germans Trias i Pujol Research Institute (Permit DAAM 9521) and has followed the principles outlined in the Declaration of Helsinki for animal experimental investigation. Human samples were obtained after the approval and in strict accordance with the recommendations of the guidelines of Germans Trias i Pujol Ethical Committee. All subjects gave written informed consent in accordance with the Declaration of Helsinki.

## Supplementary information

Supplementary Information.

## Abbreviations

T1D: Type 1 diabetes
aGLP-1: Analog glucagon-like peptide-1
NOD: Non-obese diabetic mouse
