# Supplementary material for: Antigen-specific immunotherapy combined with a regenerative drug in the treatment of experimental type 1 diabetes
Source: Sci Rep. 2020 Nov 3;10:18927. doi: 10.1038/s41598-020-76041-1 (PMC7609712; doi:10.1038/s41598-020-76041-1)
Supplement: Supplementary file 1 — Supplementary Information. [file 41598_2020_76041_MOESM1_ESM.docx]

**Antigen-specific immunotherapy combined with a regenerative drug in the treatment of experimental type 1 diabetes**

**Running title: Combined therapy for type 1 diabetes**

Adrian Villalba^1^, Silvia Rodriguez-Fernandez^1^, David Perna-Barrull^1^, Rosa-Maria Ampudia^1^, Laia Gomez-Muñoz^1^, Irma Pujol-Autonell^1^, Eva Aguilera^2^, Ruth M Risueño^3^, Mary Cano-Sarabia^4^, Daniel Maspoch^4,5^, Federico Vázquez^2^, Marta Vives-Pi*^1^

^1^ Immunology Section and ^2^ Endocrinology Section, Germans Trias i Pujol Research Institute, Autonomous University of Barcelona, Badalona, Spain

^3^ Josep Carreras Leukaemia Research Institute. Campus GTP-ICO. Badalona, Spain.

^4^ Catalan Institute of Nanoscience and Nanotechnology, CSIC and The Barcelona Institute of Science and Technology, Bellaterra, Spain

^5^ ICREA, Pg. Lluís Companys 23, Barcelona, 08010, Spain

^*^**Author for correspondence**: Marta Vives-Pi. Immunology Section. Germans Trias i Pujol Research Institute. Carretera Canyet s/n. 08916 Badalona, Spain. Phone +34935543050 ext. 6381. e-mail, [mvives@igtp.cat](about:blank)





**Supp Fig 1. Effect of Liraglutide administration in the insulitis score of non-diabetic NOD mice.** A) Total insulitis score and B) percentage of insulitis score islets in sham mice at 25 weeks of age, revealing increased insulitis and percentage of score 3 and 4 islets in mature islets when compared to neoislets. C) Total insulitis score and D) percentage of insulitis score islets in liraglutide treated mice at 25 weeks of age, revealing an increased insulitis score in neoislets and a similar percentage of score 3 and 4 islets.


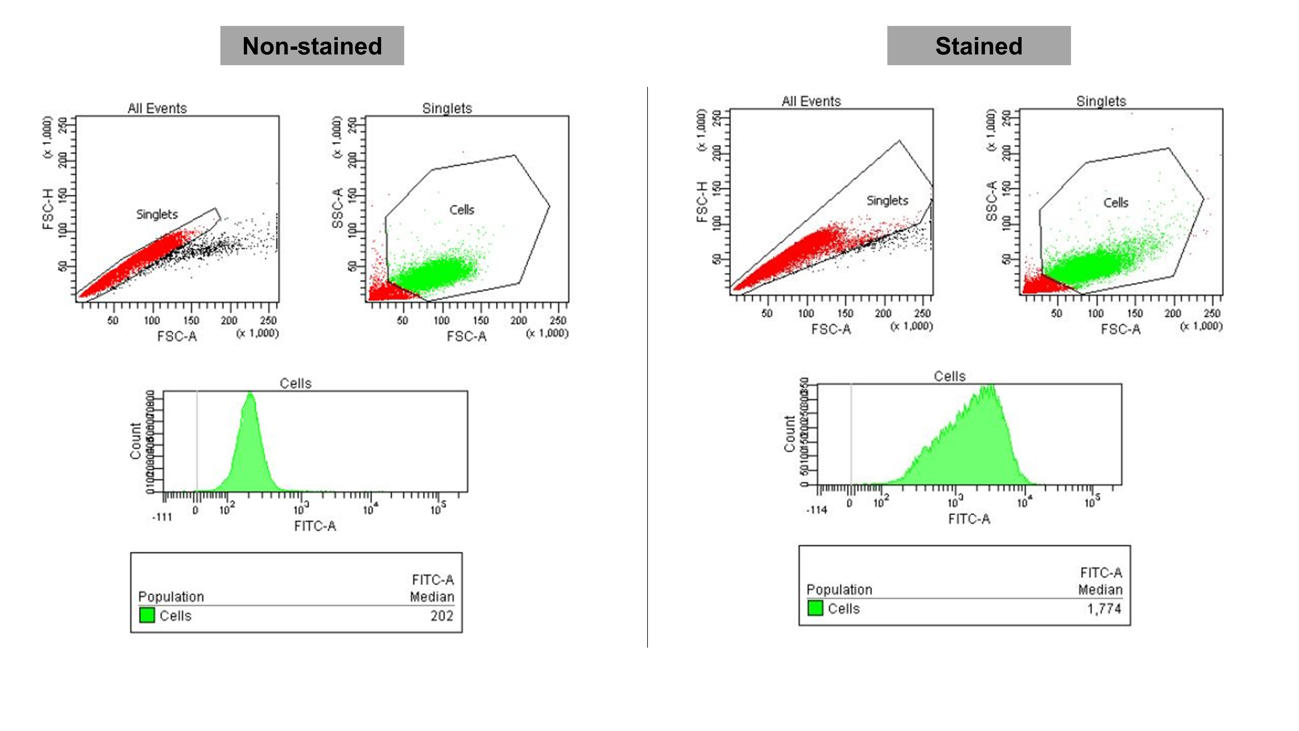


**Supp Fig 2.** Gating strategy of DCs for the phenotype assesment. The median of fluorescence intensity (MFI) was analysed from the gated cells. Non-stained and stained samples are provided to check MFI differences.
